# Evaluation of Cytochalasin B-Induced Membrane Vesicles Fusion Specificity with Target Cells

**DOI:** 10.1155/2018/7053623

**Published:** 2018-04-08

**Authors:** Marina Gomzikova, Sevindzh Kletukhina, Sirina Kurbangaleeva, Albert Rizvanov

**Affiliations:** Kazan Federal University, Kazan 420008, Russia

## Abstract

Extracellular vesicles (EV) represent a promising vector system for biomolecules and drug delivery due to their natural origin and participation in intercellular communication. As the quantity of EVs is limited, it was proposed to induce the release of membrane vesicles from the surface of human cells by treatment with cytochalasin B. Cytochalasin B-induced membrane vesicles (CIMVs) were successfully tested as a vector for delivery of dye, nanoparticles, and a chemotherapeutic. However, it remained unclear whether CIMVs possess fusion specificity with target cells and thus might be used for more targeted delivery of therapeutics. To answer this question, CIMVs were obtained from human prostate cancer PC3 cells. The diameter of obtained CIMVs was 962,13 ± 140,6 nm. We found that there is no statistically significant preference in PC3 CIMVs fusion with target cells of the same type. According to our observations, the greatest impact on CIMVs entry into target cells is by the heterophilic interaction of CIMV membrane receptors with the surface proteins of target cells.

## 1. Introduction

The discovery of extracellular vesicles in the human organism and the establishment of their role in intercellular communication lead to the development of new therapeutic approaches. EVs are 50–2000 nm in diameter membrane vesicles that are surrounded by a cytoplasmic membrane [1, 2]. It is now generally accepted that extracellular vesicles are involved in intercellular communication, delivering biologically active molecules to target cells and/or triggering receptor-mediated cellular signaling [3]. EVs are found in different human fluids including blood and lymph and are able to transport biologically active molecules to distant parts of human organism. Therefore, EVs are considered as a promising therapeutic instrument and vector for the delivery of drugs.

It was first suggested to load anticancer therapeutics into EVs for reducing chemotherapy toxicity, similar to liposomal delivery of drugs (e.g., Doxil and Myocet). To achieve targeted delivery, it was further proposed to obtain EVs from tumor cells [4]. The authors observed that EVs from tumor H22 cells were efficiently taken up by tumor H22 cells compared with primary liver cells [4]. EVs are surrounded by a cytoplasmic membrane, which protects their contents from degradation. In addition, EVs membrane receptors participate in recognition and specific binding with the surface proteins of target cells [5].

Despite the prospect of EVs application as a vector for the delivery of therapeutics, the clinical trials are few in number. The reason is that the limited amount of EVs is not sufficient for wide therapeutic use [6]. To increase the yield of vesicles surrounded by a cytoplasmic membrane, it was proposed to induce the release of membrane vesicles from the surface of human cells by treatment with cytochalasin B and application of a mechanical action, vortexing [7]. Cytochalasin B-induced membrane vesicles have functionally active surface proteins, contain the cytoplasmic component of parent cells, and maintain the reactions of cellular signaling [7]. Peng et al. [8] studied the loading of cytochalasin B-induced membrane vesicles or so-called cell membrane capsules with anticancer therapeutics rather than synthetic vehicles. They observed that membrane vesicles loaded with doxorubicin inhibit tumor growth in mouse xenografts, with significantly reduced toxicity compared to free drug [8]. More recently, it was proposed to use the cytochalasin B-induced membrane vesicles in anticancer therapy for the encapsulation of indocyanine green (ICG) [9] and methylene blue (MB) [10]. The encapsulation of ICG into cytochalasin B-induced membrane vesicles led to slowing down the body clearance of ICG and improving the effectiveness of photothermal antitumor therapy* in vivo* [9]. Vesicles loaded with MB showed lower cytotoxicity with retained photodynamic anticancer therapy effect [10].

Biocompatibility of membrane vesicles together with the enhanced yield after cytochalasin B treatment of cells makes the cytochalasin B-induced membrane vesicles (CIMVs) an attractive vector for biomolecules and/or therapeutics delivery. However, the specificity of fusion of CIMVs with target cells has not been investigated. In this regard, the aim of our work was to evaluate the fusion effectiveness of CIMVs with different types of target cells.

## 2. Materials and Methods

### 2.1. Cell Culture

PC3 (ATCC CRL1435, human prostate cancer cell line), SH-SY5Y (ATCC CRL-2266, human neuroblastoma cell line), HCT116 (ATCC CCL-247, human colorectal carcinoma cell line), and HeLa (ATCC CCL-2, human cervical cancer cell line) were grown in DMEM (Paneco, Russia) supplemented with 10% fetal bovine serum (Gibco, UK) and 2 mM L-glutamine (Paneco, Russia) at 37°С with 5% CO_2_. Cell passaging was performed using a 0.25% trypsin-EDTA solution (Life Technologies, USA).

### 2.2. CIMVs Production

CIMVs were prepared as described previously [11]. Briefly, human cells were washed twice with DPBS and incubated in DMEM containing 10 *μ*g/ml of cytochalasin B (Sigma-Aldrich, USA) for 30 min (37°C, 5% CO_2_). At the end of incubation, the cell suspension was vortexed vigorously for 30 sec and pelleted (100 g for 10 min). The supernatant was subjected to two subsequent centrifugation steps (100 g for 20 min and 2000 g for 25 min). The resulting pellet contained CIMVs.

### 2.3. Characterization of the CIMVs

*Flow Cytometry.* The size of CIMVs was determined by flow cytometry (BD FACSAria III, BD Biosciences, USA) with calibration particles (0.22-0.45-0.88-1.34-3.4 *μ*m) (Spherotech, USA).

*Dynamic Light Scattering (DLS).* The *Z*-average hydrodynamic diameters of 0.2 mg/mL CIMVs were determined at 37°C in PBS on a Zetasizer Nano ZS (Malvern, USA). Data was averaged from three parallel measurements.

### 2.4. Cell Cytoplasm and Cytoplasmic Membrane Staining

Calcein AM was used for the cell cytoplasm staining. Cells (1 × 10^6^ cells/ml) were incubated in DPBS containing 10 *μ*М of Calcein AM (eBioscience, USA) for 15 min and washed (1x) with complete medium (DMEM with 10% FBS, 2 mM L-glutamine). Lipophilic dyes DiD and DiO (Life Technologies, USA) were used to visualize cell membranes. Cell suspensions (1 × 10^6^ cells/ml) were incubated with 5 *μ*M of DiO or DiD dyes for 15 min (37°C, 5% CO_2_) and washed (2x) with complete medium (DMEM with 10% FBS, 2 mM L-glutamine). Cells and CIMVs were analyzed by confocal laser scanning microscopy (Carl Zeiss LSM 780, Germany) and flow cytometry (BD FACSAria III, USA).

### 2.5. Inhibition of Cell Surface Receptors Interaction and Endocytosis

To evaluate the input of energy-dependent endocytosis to the CIMVs internalization into the different target cells, we incubated cells at 4°С for 30 min prior to CIMVs addition and for the next 4 hours. For the direct fusion of CIMVs with the cytoplasmic membrane of target cells, the interaction of cells' surface proteins is required, which bring together and hold the interacting membranes in contact for the formation of fusion pore [12]. To evaluate the contribution of cell surface interaction to the CIMVs internalization into target cells, we incubated cells in DMEM containing 100 *μ*g/ml proteinase K for 30 min (37°C, 5% CO_2_) prior to CIMVs addition.

### 2.6. Statistical Analysis

Statistical analysis was performed using Student's *t*-test (GraphPad Software, San Diego, CA) with significance level of *p* < 0.05.

## 3. Results

### 3.1. Production of CIMVs

**Figure 1:**
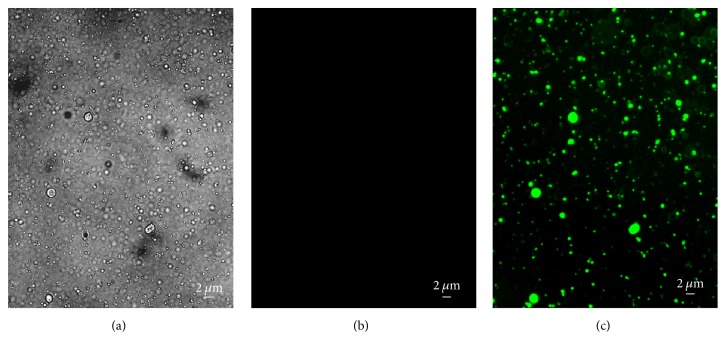
The morphology and content of CIMVs. Brightfield (a), Hoechst 33342 (b), and Calcein AM staining (c) were used to analyze the morphology and presence of nuclear and cytoplasmic components, respectively, in CIMVs.

The protocol of membrane vesicles production by cytochalasin B has been applied to many cell types in culture, including HEK293 [7, 13, 14], 3T3 fibroblast [13], HUVECC [8], and MDCKII-MDR1 [15]. We have previously reported that this protocol is suitable for HEK293 [16] and SH-SY5Y cells [11]. Here we first tested whether the protocol is suitable for PC3 cells (Figure 1). To evaluate the CIMVs integrity, we first stained the parent PC3 cells with the Calcein AM dye. This dye, after partial cleavage by intracellular esterases, converts into a fluorescent compound that is unable to diffuse through an intact cytoplasmic membrane. To confirm the absence of nuclei in the CIMVs fraction, staining with Hoechst 33342 dye was performed. We observed our CIMVs as rounded membrane-enclosed vesicles containing cytoplasmic content of parent cells without a nuclear fraction (Figures 1(b) and 1(c)).

**Figure 2:**
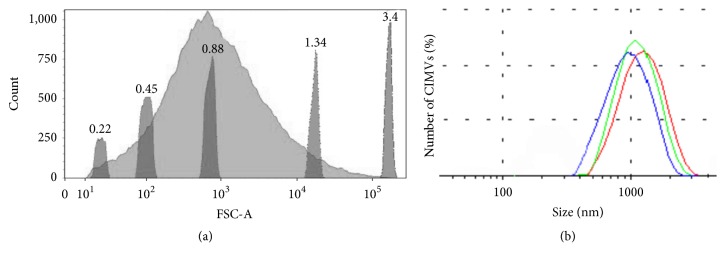
Size distribution of PC3 CIMVs. (a) Flow cytometry data. Light gray peak: distribution of PC3 CIMVs by forward-scatter (FSC); dark gray peaks: size calibration beads of 0.22, 0.45, 0.88, 1.34, and 3.4 *μ*m. (b) Dynamic light scattering data. Curves of 3 parallel measurements are represented.

CIMV's diameter varied from 220 nm to 3 *μ*m (Figure 2(a)) with the majority having a diameter of 220–1340 nm (95% of CIMVs) (Figure 2(a)). To confirm these results, we also estimated the size of CIMVs by dynamic light scattering (DLS). DLS method is fast and relatively inexpensive and has high resolution (0,3 нм – 10 *μ*м) and is widely used to determine the size of extracellular vesicles of eukaryotes and outer membrane vesicles of prokaryotes. We found that the average hydrodynamic diameter of CIMVs obtained from PC3 was 962,13 ± 140,6 nm (Figure 2(b)).

### 3.2. Fusion of CIMVs with Target Cells

Cytochalasin B-induced membrane vesicles retain cell surface receptors of the parent cells and are also able to maintain receptor-mediated signaling of the parent cell [7]. Presumably, CIMVs are able to specifically interact with target cells through the cell surface receptors [5]. Therefore, we first sought to determine the ability of CIMVs to fuse with target cells and then to determine the specificity of fusion of CIMVs with different types of target cells and whether cell surface receptors contributed to this process.

**Figure 3:**
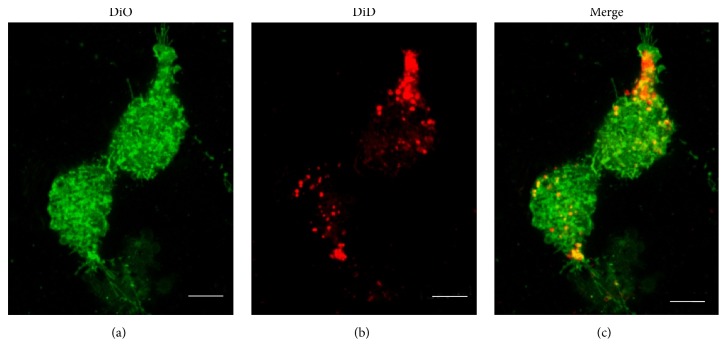
Interaction of CIMVs with recipient cells. Fluorescent microscopy (a)–(c) of PC3 recipient cells 4 h after 10 *μ*g/ml CIMVs application was carried out. Recipient cells were prelabeled with DiO cytoplasmic membrane dye and CIMVs were prelabeled with DiD membrane dye. Scale bar: 5 *μ*m.

CIMVs were obtained from the prostate cancer cell line PC3 and incubated with the PC3 target cells. To distinguish CIMVs membrane component from the cytoplasmic membrane of target cells, we performed differential staining with DiD (red fluorescence) and DiO (green fluorescence) membrane dyes, respectively. After 4 h of CIMVs incubation with PC3 cells, the CIMVs membrane component (red fluorescence) was detected in the cell membrane and in the cytoplasm of target cells at a different focal distance (Figure 3).

**Figure 4:**
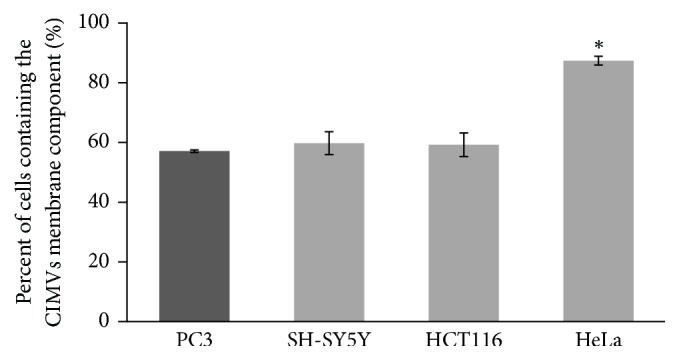
The fusion efficiency of PC3 CIMVs with PC3, SH-SY-5Y, HCT116, and HeLa cell lines (flow cytometry data). Dark gray column: PC3 CIMVs interaction with PC3 target cells (homophilic interaction). ^*∗*^*p* < 0.05 indicates significance level.

Next the efficiency of CIMVs fusion with PC3, SH-SY-5Y, HCT116, and HeLa cell lines was evaluated by flow cytometry. The fusion efficiency of PC3 CIMVs with PC3, SH-SY5Y, or HCT116 cell lines did not differ significantly (percentage of cells containing the CIMVs membrane component was 56.81 ± 0.41%, 59.46 ± 3.8%, and 58.95 ± 3.9%, resp.) (Figure 4). However, HeLa cells showed an enhanced ability to fuse with CIMVs (86.96 ± 1.46% of cells containing the CIMVs membrane component) (Figure 4).

### 3.3. Contribution of Endocytosis and Interaction of Cell Surface Receptors in CIMVs Penetration

**Figure 5:**
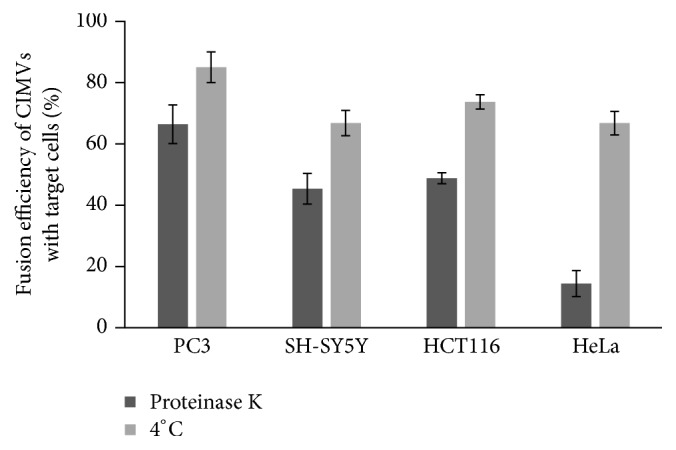
Effect of low-temperature (4°С) incubation and proteinase K treatment on fusion efficiency of CIMVs with target cells. Results are shown as percentage relative to positive control (100%, cells incubated with CIMVs at 37°C in full medium).

Previously, we have shown that CIMVs, similar to natural extracellular vesicles, penetrate into target cells by endocytosis and membrane fusion [11]. In this study, we sought to define more clearly the contribution of these processes to CIMVs penetration into different types of cells. For inhibition of energy-dependent endocytosis, we incubated target cells at 4°С. To inhibit the direct membrane fusion of CIMVs with target cells, an enzyme treatment of cells with proteinase K was carried out. Proteases disrupt the surface receptors which are required for the convergence and retention of the cytoplasmic membranes of interacting cells and CIMVs [12]. We found that decreasing temperature and proteinase K treatment effectively inhibited the CIMVs internalization into target cells (Figure 5). Incubation of cells at 4°С decreased the CIMVs internalization into PC3 cells by 15.36 ± 4.9%, SH-SY5Y by 33.5 ± 4.13%, HCT116 by 26.6 ± 2.33%, and HeLa by 33.5 ± 3.8% compared to positive control (cells incubated with CIMVs at 37°C in full medium). Proteinase K treatment decreased the CIMVs internalization into PC3 cells by 33.8 ± 6.3%, SH-SY5Y by 54.8 ± 4.97%, HCT116 by 51.4 ± 1.76%, and HeLa by 85.6 ± 4.2% compared to positive control (cells incubated with CIMVs at 37°C in full medium) (Figure 5).

The most significant decrease of CIMVs internalization was detected after the treatment of HeLa cells with proteinase K (Figure 5).

### 3.4. Heterophilic Interaction of HeLa CIMVs with Target Cells

**Figure 6:**
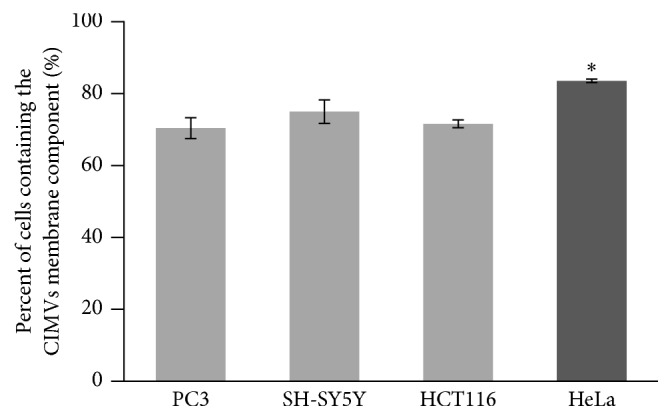
The fusion efficiency of HeLa CIMVs with PC3, SH-SY-5Y, HCT116, and HeLa cell lines (flow cytometry data). Dark gray column: HeLa CIMVs interaction with HeLa target cells (homophilic membrane proteins interaction). ^*∗*^*p* < 0.05 indicates significance level.

We found that HeLa cells demonstrated an enhanced ability to fuse with CIMVs (Figure 4). Because this phenomenon was inhibited by proteinase K treatment, we propose a heterophilic interaction of HeLa cell surface receptors. We also investigated the fusion efficiency of CIMVs from HeLa cells with different types of target cells. HeLa CIMVs demonstrated no statistically significant differences in fusion efficiency with PC3 (70 ± 2.8% of cells contain the CIMVs membrane component), SH-SY5Y (74.6 ± 3.26%), or HCT116 (71.2 ± 1.1%) cells (Figure 6) and demonstrated a little preference to fusion with the same type of cells (83.1 ± 0.48%)  (*p* < 0.01).

Thus, HeLa CIMVs demonstrated similar specificity to a wide spectrum of target cells, confirming the importance of a heterophilic interaction of the surface receptors for HeLa cells.

## 4. Discussion

Membrane vesicles act as a promising and attractive vector for biomolecules and drug delivery, since they are surrounded by a natural cytoplasmic membrane. Biocompatible membrane vesicles are not recognized as foreign and are not subjected to fast clearance [8]. The cytochalasin B application makes the procedure of isolation easier and increases the membrane vesicle yield [7]. Obtained CIMVs retain the parent cells' surface receptors [7] and are similar to natural extracellular vesicles in size and molecular content (presence of fragments of actin cytoskeleton and growth factors) [11].

According to the flow cytometry data, the size of CIMVs obtained from PC3 cells varied from 220 nm to 3 *μ*m. DLS method showed that the mean hydrodynamic diameter of obtained cytochalasin B-induced membrane vesicles was 962,13 ± 140,6 nm, providing consistency between the two techniques. It should be noted that DLS has a higher resolution and is the most widely used method for extracellular vesicle characterization. Previously, the hydrodynamic diameters evaluated for CIMVs obtained from HUVECC were 930 nm in average [8] and CIMVs obtained from human endothelial cells (ECs) were 607.3 ± 57.2 nm in PBS [9, 10]. Thus it appears that the size of CIMVs does vary depending on the type of the parent cell.

Extracellular vesicles deliver their content into recipient cells in two ways: (1) fusion of extracellular vesicles membrane with cytoplasmic membrane of recipient cells and (2) endocytosis. The first is supported by the inhibition of vesicles fusion with recipient cells after treatment of extracellular vesicles with proteinase K [17]. Proteinase K destroys surface proteins, which play an important role in the process of direct fusion of membranes. It is known that the direct fusion of membranes requires overcoming the repulsion of the negatively charged outer layer of cytoplasmic membranes. Interaction of surface proteins provides for the retention of cytoplasmic membranes and subsequent fusion [18]. A number of cell-surface proteins (such as the CAMs) mediate such retention of interacting membranes between cells of a single type, homophilic (interaction between the same molecules) adhesion, or between cells of different types, heterophilic adhesion. For example, E-cadherin and N-CAMs mediate homophilic interactions, binding together vesicles and cells that express similar molecules, whereas selectins mediate heterophilic interactions [19]. In contrast, endocytosis is supported by a decrease in the percentage of fusion of extracellular vesicles with recipient cells as a result of the application of low temperatures or dynasore, a GTPase inhibitor of endocytosis [20]. However, it remains unclear whether CIMVs possess real specificity of fusion with target cells.

The targeted delivery of therapeutics anticancer chemotherapy has the special goal of reducing toxic effects on normal cells. Prostate PC3 adenocarcinoma cells were chosen as donor cells for CIMV production due to the fact that prostate cancer is the second most common cancer in men [21]. We investigated the fusion specificity of PC3 CIMVs with different target cells (PC3, SH-SY-5Y, HCT116, and HeLa cell lines) and found that there is no statistically significant preference in CIMVs fusion with target cells of the same type (homophilic interaction). PC3-derived CIMVs did not show any specificity of fusion with PC3 target cells. However, we observed increased fusion activity of PC3 CIMVs with HeLa cells.

To investigate the contribution of endocytosis/surface proteins to CIMV uptake by target cells, we inhibited energy-dependent endocytosis/disrupted the surface receptors to investigate the role of corresponding pathways. Disruption of surface receptors had the greatest impact on penetration of PC3 CIMVs into target cells. Together, these data suggest that the greatest impact on CIMVs entry into target cells is by the heterophilic interaction of CIMVs membrane receptors with the surface proteins of target cells. The most pronounced impact of heterophilic interaction was for HeLa cells, since these demonstrated an enhanced ability to fuse with CIMVs of another cell type which was significantly inhibited by disrupting surface receptors with proteinase K.

## 5. Conclusions

Taken together, our results demonstrate that the use of membrane vesicles obtained from the same type of cells is unlikely to achieve targeted delivery, since surface receptors have wide specificity. We found that a heterophilic interaction of CIMVs membrane receptors with the surface proteins of target cells was the most important interaction for successful fusion.
